# The use of deformation imaging in the assessment of patients pre and post transcatheter aortic valve implantation

**DOI:** 10.1186/s44156-023-00017-w

**Published:** 2023-02-22

**Authors:** Mark Coyle, Gerard King, Kathleen Bennett, Andrew Maree, Mark Hensey, Stephen O’Connor, Caroline Daly, Gregory Murphy, Ross T. Murphy

**Affiliations:** 1 Department of Cardiology, St James Hospital, Dublin, Ireland; 2 Institute of Cardiovascular Science, St James Hospital, Dublin, Ireland; 3 School of Population Health, Royal College of Surgeons Ireland, University of Medicine and Health, Dublin, Ireland

**Keywords:** Aortic stenosis, TAVI, Heart failure, LV strain, Reduced ejection fraction, Valve disease

## Abstract

**Background:**

Deformation imaging represents a method of measuring myocardial function, including global longitudinal strain (GLS), peak atrial longitudinal strain (PALS) and radial strain. This study aimed to assess subclinical improvements in left ventricular function in patients undergoing transcatheter aortic valve implantation (TAVI) by comparing GLS, PALS and radial strain pre and post procedure.

**Methods:**

We conducted a single site prospective observational study of 25 patients undergoing TAVI, comparing baseline and post-TAVI echocardiograms. Individual participants were assessed for differences in GLS, PALS and radial strain in addition to changes in left ventricular ejection fraction (LVEF) (%).

**Results:**

Our results revealed a significant improvement in GLS (mean change pre-post of 2.14% [95% CI 1.08, 3.20] p = 0.0003) with no significant change in LVEF (0.96% [95% CI − 2.30, 4.22], p = 0.55). There was a statistically significant improvement in radial strain pre and post TAVI (mean 9.68% [95% CI 3.10, 16.25] p = 0.0058). There was positive trend towards improvements in PALS pre and post TAVI (mean change of 2.30% [95% CI − 0.19, 4.80] p = 0.068).

**Conclusion:**

In patients undergoing TAVI, measuring GLS and radial strain provided statistically significant information regarding subclinical improvements in LV function, which may have prognostic implications. The incorporation of deformation imaging in addition to standard echocardiographic measurements may have an important role in guiding future management in patients undergoing TAVI and assessing response.

## Introduction

Aortic stenosis (AS) is the commonest primary valvular abnormality necessitating surgical or percutaneous intervention in Europe and North America [1]. The most common cause of AS is senile calcification and consequently the prevalence of this valvular abnormality is increasing worldwide as the population ages and life expectancy increases [2]. AS leads to compensatory anatomical and physiological changes in the left ventricle (LV) culminating in detrimental effects including left ventricular hypertrophy (LVH), diastolic dysfunction and ultimately LV failure [3]. Operative treatment options for severe AS include surgical aortic valve replacement (SAVR) and transcatheter aortic valve implantation (TAVI). Since its introduction in 2002, TAVI has progressed and developed in recent years as a treatment option for AS in elderly patients. The most recent European Society of Cardiology (ESC) guidelines for valvular heart disease published in 2021 recommend that all patients over 75 years of age, or those unsuitable or too high-risk for SAVR should be considered for TAVI [4]. TAVI has been shown to improve symptoms, reduce progression of LV dysfunction and ultimately reduce mortality [5].

Transthoracic echocardiography (TTE) remains the gold standard investigation of choice for diagnosing and quantifying the severity of AS with several echocardiographic parameters being used. Historically, left ventricular ejection fraction (LVEF) % has been the most commonly used measure to determine LV systolic function. More recently however deformation imaging has become an important method of determining clinical and subclinical assessment of heart chamber function. Deformation imaging encompasses myocardial strain such as shortening, thickening and lengthening of myocardial fibres and includes global longitudinal strain (GLS), peak atrial longitudinal strain (PALS) and radial strain [6]. GLS is an important echocardiographic parameter that may be used as a predictor of cardiovascular outcomes and subclinical changes in LV function.

Deformation imaging also allows us to assess left atrial function and also can assess the different phases of left ventricular filling dynamics. In the left atrial (LA) reservoir phase, as the LA fills and stretches, there is deformation called peak left atrial longitudinal strain (PALS), which peaks in systole at the end of LA filling, before the opening of the mitral valve, and is reflective of LV systolic function. Radial left ventricular strain represents the sum of both subepicardial and subendocardial radial deformations, as there are no radial myocyte fibres. Incorporating GLS, PALS and global radial strain (GRS), in addition to standard echocardiographic measurements, may be useful as a predictor of cardiovascular outcomes in patients post TAVI insertion.

The primary aim of this study is to assess changes in GLS, PALS and GRS in patients pre and post-TAVI as predictors of subclinical improvements in LV function. Our secondary aim is to provide information for the development of future larger scale studies.

## Methods

### Study population

This study was a single site prospective observational study in St. James Hospital, Dublin, Ireland. A total of 25 patients were included in the study. Patients were identified from hospital lists and from the hospital’s cardiology outpatient department. Pre-TAVI echocardiograms were performed as routine work-up before valve implantation. Patients were recalled for out-patient review and echocardiogram examination approximately 6 weeks post-TAVI. Some patients did not have local follow-up and therefore were contacted to present for an out-patient echocardiogram approximately 6 weeks post-TAVI. There were 11 individuals with post-TAVI TTEs performed within 14 days of their procedure due to difficulty attending 6 week follow-up. The mean (SD) interval before pre-TAVI TTE was 25.84 (28.2) days; median = 16 (IQR 6, 32) days. The mean (SD) interval before post-TAVI TTE was 84.8 (138.9) days; median 26 (3, 45) days.

### Data collection

Standard trans-thoracic echocardiograms (TTE) were performed by trained British Society of echocardiography accredited cardiac physiologists using GE Healthcare machines. All standard echocardiography views were recorded. Captured images were subsequently downloaded onto discs and further analysed at a later date.

### Data analysis

Echocardiograms were analysed off-line using EchoPAC™ software (GE Vingmed Ultrasound AS Horten Norway. Version 0.201). Analysis was performed by a single blinded operator who was an experienced cardiac research physiologist. Echocardiogram measures were calculated according to British Society of Echocardiography specifications. Ejection fraction was calculated using Simpson`s biplane. The transmitral flow and isovolumic relaxation time (IVRT) were recorded by Doppler ultrasound. The E/A ratio was computed as a ratio of peak velocity in the early rapid filling phase when the ventricle relaxes (E wave) and peak velocity of the late filling due to atrial contraction (A wave) across the mitral valve during diastole. The myocardial performance index (Tei) was derived from the mitral valve closure to opening time and the left ventricle (LV) ejection time. LV mass was determined from septal and posterior wall thicknesses and LV end diastolic diameter and indexed to body surface area. Myocardial strain—the change in myocardial fibre length over the cardiac cycle—is a measure of myocardial muscle function that can be obtained using conventional assessment techniques and is an earlier marker of myocardial disease than ejection fraction. It was measured by tissue tracking analysis in the apical four chamber view and the parasternal short axis view centred on the left ventricle. All analysed segments were approved by both the programme and the operator. To assess for inter-observer variability, a second BSE accredited physiologist analysed 10 studies and results were compared. Krippendorff’s alpha intercoder reliability coefficient was 0.97 for the inter-rater reliability demonstrating very strong reliability between raters.

### Outcome measures

Individual changes in GLS, PALS and GRS and subclinical improvements in LV function pre- and post-procedure were compared. PALS was derived from longitudinal strain curves generated for each of six atrial segments, obtained from the apical four chamber and two chamber views. GRS was averaged from regional segments as circumferential strain using the PSAX views at MV and PM level. Measurements of heart rate, left ventricular ejection fraction, left ventricular mass index, left ventricular dimension, left atrium dimension and E/e′ were incorporated. All analyses were performed on the GE Echo Pac v204 software.

### Ethical approval

Ethical approval was granted by the local ethics committee, St James Hospital/Tallaght University Hospital Joint Research Ethics Committee (011006/16801). All participants in the study provided written informed consent to participate in the study.

### Statistical analysis

Descriptive analysis includes means (with standard deviation, SD) for continuous data and frequencies/percentages for categorical data. Paired t-test is used to compare changes pre- and post-valve implantation. All statistical analyses were carried out with Stata v17.0. Significance at p < 0.05 is assumed.

## Results

### Demographics

A total of 25 participants were included in this study, 76% were male (n = 19) and 24% (n = 6) were female. The mean age was 80.64 (SD 8.63). Patient characteristics are shown in Table 1.

**Table 1:** Patient characteristics

Characteristic	Number % (n)
Hypertension	76% (19)
Hyperlipidaemia	48% (12)
Diabetes	32% (8)
IHD	48% (12)
AF	36% (9)
Antihypertensive medication	84% (21)
Antiplatelet agent	56% (14)
Statin	72% (18)

*N* number, *IHD* ischaemic heart disease, *AF* atrial fibrillation

### Echocardiographic outcomes

**Fig. 1:**
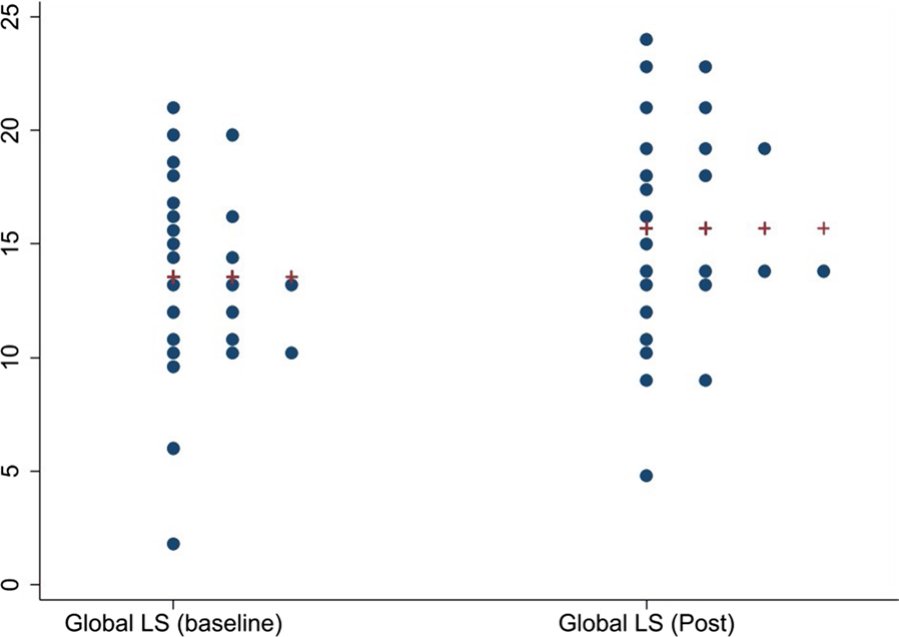
Distributional dotplots (individual data) for Global Longitudinal Strain

**Fig. 2:**
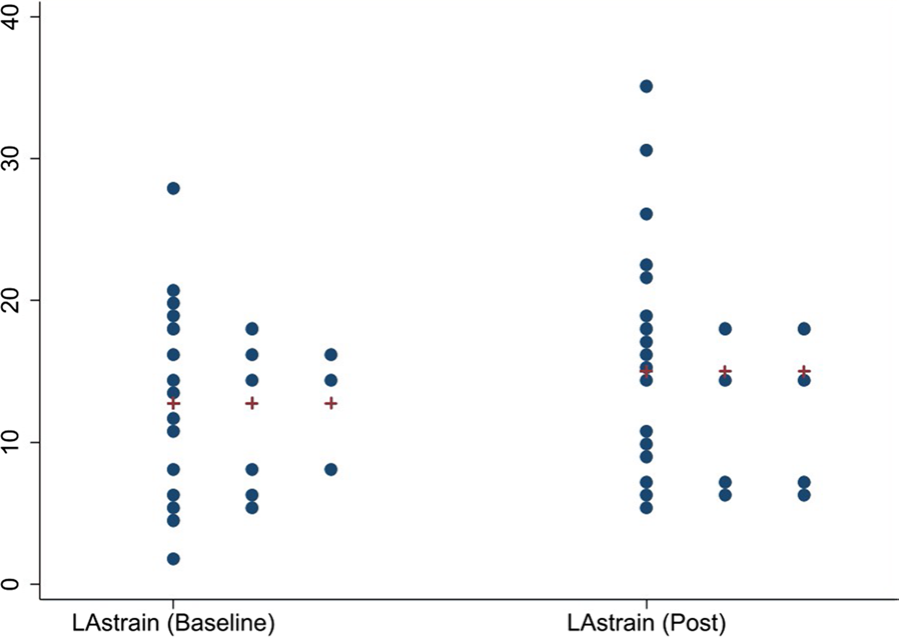
Distributional dotplots (individual data) for LA Strain

**Fig. 3:**
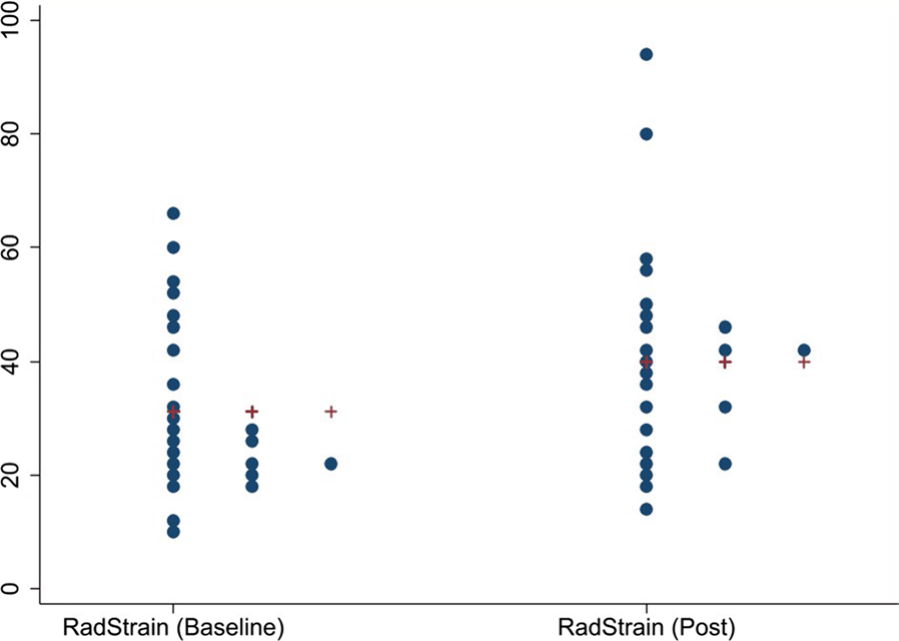
Distributional dotplots (individual data) for Radial Strain

Our results revealed a significant improvement in GLS (mean change (post–pre) of 2.14% [95% CI 1.08, 3.20] (p = 0.0003) with no significant change in ejection fraction (0.96 [95% CI − 2.30, 4.22] (p = 0.55). Although there was a positive trend toward improvement in PALS, there was no significant difference post-TAVI and this reflected basic ejection fraction (EF) measurements which remained the same, mean change (post–pre) was 2.30% (95% CI − 0.19, 4.80), p = 0.068. The mean change in radial strain was − 9.68% (95% CI − 16.25, − 3.10), p = 0.0058. Descriptive statistics pre- and post-TAVI are shown in Table 2. Figures 1, 2 and 3 show individual changes in GLS, PALS and radial strain, respectively. There were no significant differences in outcomes in those with early post-TAVI TTE compared to later post-TAVI TTE.

**Table 2:** Descriptive statistics for pre- and post-TAVI values of global longitudinal strain, peak atrial longitudinal strain, radial strain and left ventricular ejection fraction

Variable	Pre-TAVI mean (SD)	Post-TAVI mean (SD)	Difference (95% CI) post–pre	p-value
GLS (n = 25)	13.55 (4.46)	15.69 (4.88)	2.14 (1.08, 3.20)	0.0003
PALS (n = 24)	12.76 (6.42)	15.07 (8.15)	2.3 (− 0.19, 4.80)	0.068
Radial Strain (n = 23)	30.19 (14.95)	39.87 (19.42)	9.68 (− 16.25, − 3.1)	0.0058
LVEF (n = 25)	54.32 (15.59)	55.28 (13.63)	0.96 (− 2.30, 4.22)	0.55
E prime (n = 9)	5.56 (20.0)	7.0 (2.65)	1.44 (2.92)	0.18
E/e (n = 8)	16.05 (9.2)	13.13 (6.44)	− 2.93 (5.53)	0.18
E/A (n = 17)	0.80 (0.27)	0.77 (0.28)	− 0.03 (0.28)	0.71

*TAVI* transcatheter aortic valve implantation, *CI* confidence interval, *GLS* global longitudinal strain, *PALS* peak atrial longitudinal strain, *LVEF* left ventricular ejection fraction

## Discussion

Our findings show that TAVI led to significant improvements in both GLS and radial strain. In participants undergoing TAVI, measuring radial strain provided important prognostic information regarding improvements in LV function. Measuring PALS did not provide any statistically significant difference post-procedure. These findings are significant, as improvements in GLS have been shown to be inversely correlated with mortality in patients with reduced LV function and are a predictor of deterioration in cardiac function, future heart failure events and all-cause mortality [7–9]. Furthermore, a systematic review and meta-analysis of the prognostic implications of GLS and EF found that there was strong evidence for the prognostic benefit of GLS and it appeared to have an even superior prognostic value to EF for predicting major adverse cardiovascular events [10].

Our findings are comparable to those of other similar studies. GLS appears to have a significant role but the significance of PALS and radial strain has been more ambiguous. These findings have been replicated in the short, medium and longer term post-TAVI. In the short term, a German study of 30 patients with severe AS undergoing TAVI showed significant improvements in GLS, with no changes in radial or circumferential strain and no difference in LVEF in participants comparing baseline echo to their echo one week post procedure [11]. Similarly Cimino et al. demonstrated acute changes in GLS following TAVI five days post procedure, examining separate layers of the myocardium showing significant improvements in endocardial layer LS in those with concentric LVH compared to those with eccentric hypertrophy [12]. Equivalently, in a US population of 109 patients, thirty days post TAVI there was significant short-term reverse LV and LA remodelling, with improvements in LV GLS and all three components of LA phasic function, without any recovery of ejection fraction [13]. Early improvements in GLS were also shown in a United Kingdom TAVI population over a mean follow-up period of 49 days and corresponding with previous studies this was without any change in LVEF [14].

Other investigations have shown a more protracted time to accrue this benefit, in 150 patients undergoing TAVI in Germany, baseline GLS was similar to one week post TAVI but showed significant improvements at 3 months without any changes in circumferential strain or GRS. Interestingly, GLS was also directly correlated with post-procedural outcomes [15]. Furthermore, GLS has been shown to be directly linked to perioperative outcomes, with a higher adverse event rate in those with changes in GLS during their procedure [16]. An analysis of patients undergoing TAVI in Italy comparing 2D strain speckle analysis pre-TAVI, immediately post-TAVI and 3 months post-TAVI, revealed immediate improvements in 2D strain post-procedure with continued improvements at 3 months. There were no improvements in the immediate phase in either radial or circumferential strain but there were improvements in both at 3 months. It was postulated that the early reduction in LV longitudinal strain could represent a quick response to acute reduction in afterload post-TAVI [17].

Focusing on longer-term outcomes, these changes in strain have been shown to be associated with improved individual outcomes. Within 6 months of TAVI there was reverse LA remodelling and an improvement in LA reservoir function [18]. In an Italian heart population, 6 months post-TAVI GLS significantly improved and consequently led to reductions in LV mass [19]. However, despite improvements in both anatomy and functionality of the LV, similar to our findings, limited improvements in LA strain both in the early phase post-TAVI and at 6 months follow-up have been shown in a post-trial analysis of the RETORIC trial in Hungary [20].

The long-term sustained improvement in GLS post-TAVI has been shown in two studies of patients followed up a mean of 8 months post implantation with improvements in all three of strain, strain rate and twist, as well as in a population followed up for one year post-TAVI [21, 22]. Similarly, analysis of the SOURCE 3 registry of 16 European centres examining changes in pre procedure, post procedure and one year follow-up, GLS improved significantly and this was sustained at one year with no significant change in LVEF [23]. It is highly important to document improvements in LV function considering the high prevalence of concomitant cardiac amyloidosis (CA) in this population. The prevalence of CA in patients with AS increases in prevalence in the older population and confers a poor prognosis, as has been described from early insights into the CAMPOS-TAVI trial [24]. However, a recent systematic review and meta-analysis indicated that there is a significant benefit in aortic valve replacement (SAVR or TAVI) in this specific population [25].

### Implications for clinical practice

Our results show that use of deformation imaging to measure myocardial strain can lead to detection of subclinical changes in LV function which may be a predictor of patient prognosis post-TAVI. These findings are important, as from previous studies changes in GLS have been shown to be directly associated with clinical outcomes. Therefore, incorporating deformation imaging as adjunctive to standard echocardiographic measurements may provide additional information to risk-stratify patients based on LV function.

## Conclusion

In participants undergoing TAVI, measuring GLS provided important information regarding improvements in LV function which may have prognostic implications. GLS appears to have a significant role in predicting prognosis in patients undergoing TAVI. Measuring GLS may potentially have an important role in the management of this cohort of patients in the future, when coupled with the routine echocardiographic examination. In participants undergoing TAVI, measuring PALS may only be reflective of conventional measurements of systolic left ventricular function such as EF and may not reflect subclinical findings. Radial strain appears to have a significant role in predicting prognosis in patients undergoing TAVI. Measuring radial strain may potentially have an important role in the future management of this cohort of patients, in addition to the routine echocardiographic examination.

## Limitations

This study was limited by a short follow-up period of 6 weeks. A longer follow-up period examining these echocardiographic parameters would be preferable to determine longer term changes in strain and LVEF. This study was also limited by varying follow up times between patients. An additional limitation was that blood pressure was not recorded at the time of participant TTE. Recommendations for future research would be to enrol a larger number of participants over a greater follow-up period, with incorporation of participant blood pressure at the time of TTE.

## Data Availability

The dataset supporting the conclusions of this article are available from the corresponding author on reasonable request.
